# An Account of a Remarkable Tumor on the Head Opened and Cured

**Published:** 1785

**Authors:** 


### III. *An Account of a remarkable Tumor on the Head opened and cured.* *By* the ſame.

RICHARD Murriſh, of the pariſh of Madron, in Cornwall, aged fifty years, in the ſummer of 1782 complained of a ſlight pain in the left parietal bone, which daily increaſed, ſo as to confine him to his bed, when a collection of matter took place juſt below the maſtoid proceſs on the ſame ſide, and the abſceſs continued for ſeveral months to yield a thin purulent diſcharge, without giving the leaſt relief to the diſtracting pain which he now felt over the left ſide of his head.

On the 23d of December following, when I firſt viſited him, his weakneſs, from pain and want of reſt, ſcarcely permitted him to ſit up in bed; his appetite was quite loſt; he had profuſe night ſweats, with violent ſhiverings; and his body was greatly emaciated.

He deſcribed the pain as extending over the whole of the left parietal bone, and ſaid, he felt as if a cord was drawn exceedingly tight round his head.

On examining the ſcalp, a diffuſed tumor, without any ſenſible fluctuation, appeared all over the parietal bone, and, on being preſſed,

 gave gave him great uneafinefs. The wound juft below the ear was fmall, yielded good matter, and admitted, through a quantity of fungous flefh, a probe two or three inches within the cranium.

From the appearance of the fcalp, fufpecting that matter was formed under the pericranium, I made an opening of three inches in length on the fuperior part of the left parietal bone; the fcalp was grown to the amazing thicknefs of an inch and a half, and under the pericranium a large quantity of good pus was collected, fo that the fkull was laid bare over the greateft part of the parietal bone. On the next day my patient was much altered for the better, having flept well all night, and the pain being greatly abated.

On examining the fkull, we now difcovered a perforation in the center of the parietal bone, juft large enough to admit a probe, which paffed entirely through the cranium, and, by making the aperture a little bloody, produced a fimilar appearance in the wound at the proceffus maftoideus; fo that the difcharge from the latter was evidently fupplied by the matter that infinuated itfelf through the opening in the parietal bone, and paffed from thence, between the dura mater and cranium, to the aperture below.

After a few days the wound below healed, and, in three weeks, the denuded cranium was covered with fine granulations, without any exfoliation; and in a fortnight more this wound alſo was completely healed, and the patient's health perfectly reſtored.

This man did not remember to have received a blow on this part, nor could we attribute this peculiar appearance to any diſeaſe of habit. He ſtill continues well, and without any complaint in that part of his head.

